# Posttranslational modification of a vanadium nitrogenase

**DOI:** 10.1002/mbo3.265

**Published:** 2015-06-19

**Authors:** Erin K Heiniger, Caroline S Harwood

**Affiliations:** Department of Microbiology, University of WashingtonSeattle, Washington, 98195

**Keywords:** Hydrogen production, nitrogen fixation, *Rhodopseudomonas palustris*

## Abstract

In microbes that fix nitrogen, nitrogenase catalyzes the conversion of N_2_ to ammonia in an ATP-demanding reaction. To help conserve energy some bacteria inhibit nitrogenase activity upon exposure to ammonium. The purple nonsulfur phototrophic bacterium *Rhodopseudomonas palustris* strain CGA009 can synthesize three functional nitrogenase isoenzymes: a molybdenum nitrogenase, a vanadium nitrogenase, and an iron nitrogenase. Previous studies showed that in some alphaproteobacteria, including *R. palustris*, molybdenum nitrogenase activity is inhibited by ADP-ribosylation when cells are exposed to ammonium. Some iron nitrogenases are also posttranslationally modified. However, the posttranslational modification of vanadium nitrogenase has not been reported. Here, we investigated the regulation of the alternative nitrogenases of *R. palustris* and determined that both its vanadium nitrogenase and its iron nitrogenase activities were inhibited and posttranslationally modified when cells are exposed to ammonium. Vanadium nitrogenase is not found in all strains of *R. palustris*, suggesting that it may have been acquired by horizontal gene transfer. Also, phylogenetic analyses of the three nitrogenases suggest that VnfH, the target of ADP-ribosylation, may be the product of a gene duplication of *nifH*, the molybdenum nitrogenase homolog.

## Introduction

Many bacteria and archaea can convert N_2_ to ammonia, a bio-available form of nitrogen that sustains life on earth (Igarashi and Seefeldt 2003). The energetically demanding reaction of reducing the triple bond of N_2_ to ammonia is accomplished by the highly conserved enzyme molybdenum (Mo^−^) nitrogenase with the following stoichiometry: N_2_ + 8e^−^ + 8H^+^ + 16ATP → 2NH_3_ + H_2_ + 16ADP (Burris 1991; Igarashi and Seefeldt 2003). Although the main function of nitrogenase is the production of ammonia, it also produces H_2_ as a product. In the absence of N_2_, nitrogenase reduces protons exclusively, forming pure H_2_, a process that can potentially be exploited for the biological production of hydrogen fuel (McKinlay and Harwood 2010; Keskin et al. 2011). *Rhodopseudomonas palustris* is a purple nonsulfur photosynthetic bacterium that has served as a model organism for biological H_2_ production via nitrogenase (McKinlay and Harwood 2010). It can generate the ATP needed for the energy-intensive nitrogenase reaction from the abundant resource of sunlight and it can degrade structurally diverse organic compounds, including lignin monomers that are typically found in agricultural waste, as a source of electrons for H_2_ production (Harwood and Gibson 1988; Barbosa et al. 2001; Huang et al. 2010; Gall et al. 2013). From a bioengineering standpoint, *R. palustris* is a hardy organism that can produce H_2_ continuously for months without significant loss of viability (Gosse et al. 2007).

*R. palustris* strain CGA009 synthesizes alternative iron (Fe^−^) and vanadium (V^−^) nitrogenases, encoded by *anfHDKG* and *vnfHDGK* genes, in addition to Mo-nitrogenase (Larimer et al. 2004; Oda et al. 2005). The alternative enzymes differ from Mo-nitrogenase in the transition metal present at the active site (Bishop et al. 1980; Burris 1991; Eady 1996). They are synthesized and active in situations where Mo becomes limiting. In addition to being less prevalent in microbes, the alternative nitrogenases are also less efficient for nitrogen fixation, consuming more reducing power and producing more H_2_ per molecule N_2_ fixed than Mo-nitrogenase (Eady 1996). This makes them good candidates for potential use in a H_2_ production process.

Because nitrogen fixation is an energetically demanding and slow process and because the synthesis of nitrogenase is very complex, its synthesis tends to be strongly repressed by ammonium (Dixon and Kahn 2004). In some bacteria nitrogenase activity is also controlled posttranslationally by reversible ADP-ribosylation at a conserved arginine on dinitrogenase reductase (NifH or AnfH) (Pope et al. 1985; Lowery et al. 1986; Masepohl et al. 1993). *R. palustris* inactivates its Mo-nitrogenase posttranslationally in response to ammonium exposure using the ADP-ribosyltransferase enzyme, DraT2 (Heiniger et al. 2012). Here, we explored whether the alternative nitrogenases of *R. palustris* are also inactivated by posttranslational modification. We were especially interested in the V-nitrogenase, as this isozyme is not known to be posttranslationally modified. We also explored the evolutionary relationships of the nitrogenase isozymes and present evidence that VnfH is the product of a gene duplication event.

## Materials and Methods

### Bacterial growth

*R. palustris* strains used in this study (Table1) were grown anaerobically in light at 30 ± 2°C in sealed tubes in nitrogen-fixing (NF) medium, a nitrogen-free mineral-based minimal medium with N_2_ in the headspace, for NF conditions (Oda et al. 2005). NF medium was supplemented with 20 mmol/L sodium acetate, 0.3% yeast extract, 1 mmol/L VCl_3_, and 1X Wolfe’s vitamins (Kim and Harwood 1991; Kieft et al. 1999).

**Table 1:** *Rhodopseudomonas palustris* strains used in this study

Strain	Genotype or phenotype	Reference
CGA009	Wild-type strain; spontaneous Cm^r^ derivative of CGA001	Harwood and Gibson (1988)
CGA753	Δ*vnfH*, Δ*anfH*, Mo-nitrogenase strain, derivative of CGA009	Oda et al. (2005)
CGA755	Δ*nifH* Δ*vnfH*; Fe-nitrogenase strain, derivative of CGA009	Oda et al. (2005)
CGA766	*ΔnifH, nifD::*Tn5, Δ*anfA*, V-,nitrogenase strain, derivative of CGA009	Oda et al. (2005)

### Hydrogen assays

Nitrogenase activity was monitored by the accumulation of H_2_ in the headspace of the cultures. Cultures of *R. palustris* were allowed to grow to an OD_660_ of 0.35–0.45. Cells (30 mL) were harvested anaerobically and resuspended in 10 mL 25 mmol/L sodium phosphate/potassium phosphate buffer, pH 7.0. Cells were transferred to sealed 27 mL tubes containing an argon atmosphere and allowed to recover for 60 min before H_2_ was measured by gas chromatography as previously described (Oda et al. 2005). H_2_ production was established for approximately 20 min before either sodium chloride or ammonium chloride was added to the assay tube to a final concentration of 100 *μ*mol/L. Stocks of 10 mmol/L sodium chloride and ammonium chloride were prepared and stored anaerobically. H_2_ produced was normalized to total cellular protein. The protein content of cell suspensions was estimated from the OD_660_ of the cell culture using a standard curve prepared with whole *R. palustris* CGA009 cells grown under NF conditions. To generate this curve, the Bio-Rad (Hercules, CA, USA) Protein Assay was used to measure total protein from NaOH lysed cells (Bradford 1976).

### Protein modification assay

Protein for visualization of posttranslational modification of dinitrogenase reductase was prepared from cells that had been grown under NF conditions as previously described (Heiniger et al. 2012). Low cross-linker sodium dodecyl sulfate polyacrylamide gel electrophoresis (SDS-PAGE) gels were used to resolve the modified protein from the unmodified protein (acrylamide: bisacrylamide ratio was 171:1). Proteins were transferred to a PVDF membrane and incubated with rabbit antiserum prepared against NifH purified from *Azotobacter vinelandii*. Antibodies to NifH cross-reacted with protein of the same molecular mass as VnfH and AnfH, although a 10-fold higher concentration of antibody was required to visualize AnfH than either VnfH or NifH. Anti-rabbit horseradish peroxidase secondary antibody was hybridized to the primary antibody and Pierce/ThermoFisher (Waltham, MA, USA) ECL femto-substrate was used for visualization.

### Protein alignment and phylogenetic tree generation

Protein sequences were accessed from the GenBank database and input in the ClustalW alignment tool (Larkin et al. 2007; Benson et al. 2012). Phylogenetic trees were built using Mega5 software (Tamura et al. 2011). Protein sequences were aligned using the ClustalW algorithm and phylogenies inferred using the neighbor-joining method after all alignment gaps were deleted (Saitou and Nei 1987; Larkin et al. 2007). The bootstrap consensus trees inferred from 500 replicates were taken to represent the evolutionary history of the taxa analyzed (Felsenstein 1985). Branches corresponding to partitions reproduced in less than 50% bootstrap replicates were collapsed. The evolutionary distances were computed using the p-distance method and are in the units of the number of amino acid differences per site (Nei and Kumar 2000).

## Results

**Figure 1:**
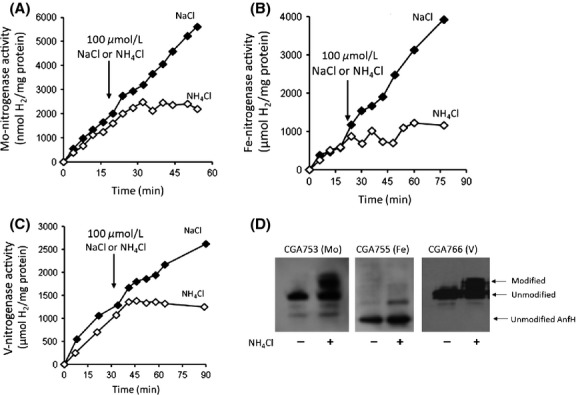
All three nitrogenase isozymes are subjected to posttranslational inactivation. (A) H_2_ produced over time by the Mo-nitrogenase expressing strain CGA753. At the arrow, either NaCl (closed symbols) or NH_4_Cl (open symbols) was added to the cell suspension at a final concentration of 100 *μ*mol/L. The data shown are one representative experiment of at least four experiments, all of which showed similar results. (B) H_2_-production switch-off of the Fe-nitrogenase-only expressing strain CGA755. (C) H_2_-production switch-off of the V-nitrogenase-only expressing strain CGA766. (D) Anti-NifH immunoblot of protein harvested from cells exposed for 30 min to either NaCl (−) or NH_4_Cl (+). The unmodified forms of NifH and VnfH run with the 32 kDa size marker, whereas unmodified AnfH runs at 30 kDa.

**Figure 2:**
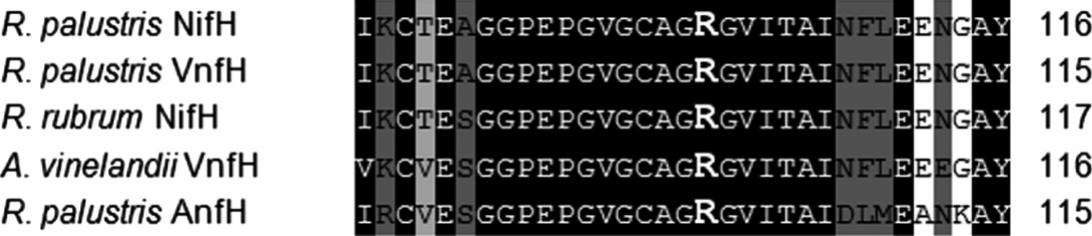
Clustal W alignment of five nitrogenase reductase proteins in the region encompassing the R101 that is the site of ADP-ribosylation on the NifH protein from *Rhodospirillum rubrum*. Also included are NifH, VnfH, and AnfH encoded by the *Rhodopseudomonas palustris* genome as well as the VnfH protein of *Azotobacter vinelandii*DJ. The residue shown to be modified in the *R. rubrum* protein, Arginine 101, is in bold (Oda et al. 2008).

We previously reported that Mo-nitrogenase activity is inhibited by the addition of ammonium to suspensions of wild-type cells (Heiniger et al. 2012). In that study, nitrogenase activity was assayed by the commonly used acetylene reduction assay. However, acetylene is a poor substrate for V- and Fe-nitrogenases. Therefore, we measured H_2_ production as a proxy for nitrogenase activity. When we did so, we found that ammonium chloride addition inhibited H_2_ production by cell suspensions prepared from *R. palustris* mutants that expressed only Mo-nitrogenase, only Fe-nitrogenase or only V-nitrogenase (Oda et al. 2005). This suggests that the dinitrogenase reductase subunits of each of the three nitrogenases may be posttranslationally modified (Fig.1, Table2). It is known that arginine101 of the *Rhodospirillum rubrum* NifH protein is ADP-ribosylated (Ma and Ludden 2001). Protein alignments showed that the NifH, VnfH, and AnfH dinitrogenase reductase proteins from *R. palustris* also have a conserved arginine at this position (Fig.2).

**Table 2:** Fe-nitrogenase is incompletely inactivated after ammonium addition

Strain	Genotype	Percent activity remaining (SEM)^1^	*P*-value
CGA753 (Mo)	Δ*anfH* Δ*vnfH*	4 (3)	0.05
CGA755 (Fe)	Δ*nifH* Δ*vnfH*	35 (12)	0.002
CGA766 (V)	*nifD*::Tn5 Δ*nifH* Δ*anfA*	1 (1)	0.02

1 Value is the percent activity remaining after NH_4_Cl addition, averaged over four experiments. The standard error of the mean is shown in parentheses.

We carried out immunoblot analysis and found that the dinitrogenase reductase proteins from the Mo-nitrogenase-only, V-nitrogenase-only, and Fe-nitrogenase-only mutant strains were modified when cells were exposed to ammonium, as evidenced by the appearance of a slower-migrating form of these proteins on SDS-PAGE gels. Full inactivation of NifH (and presumably VnfH and AnfH) occurs when 50% of the subunits are modified, as NifH is found as a dimer and modification of one subunit sterically blocks modification of the second subunit (Lowery et al. 1986). The vanadium nitrogenase VnfH is 94% identical and 98% similar to NifH, whereas AnfH is only 58% identical and 75% similar to NifH. Also AnfH is slightly smaller than NifH and VnfH. Although both alternative nitrogenase reductase proteins were modified after ammonium addition, the degree of modification of AnfH tended to be less than that of NifH and VnfH (Fig.1D). Consistent with this, we found that on average the Fe-nitrogenase-only strain did not lose as much H_2_ production activity as the Mo- and V-nitrogenase expressing strains upon exposure to ammonium (Table2).

**Figure 3:**
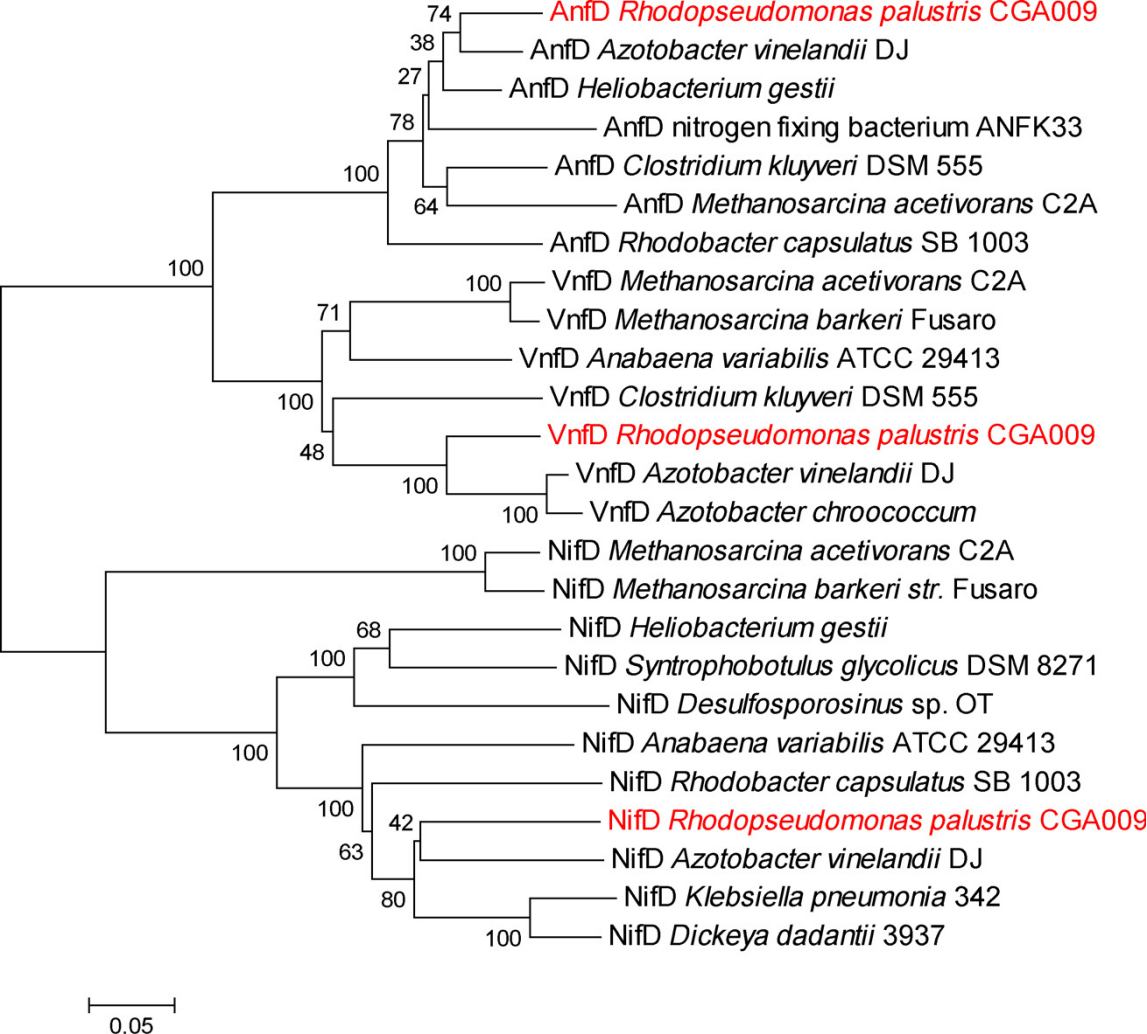
Evolutionary relationships of all three nitrogenase isozymes show separate phylogenies for each type of nitrogenase. The evolutionary histories of NifD, VnfD, and AnfD were inferred using the neighbor-joining method as described in Materials and Methods. The percentage of replicate trees in which the associated taxa clustered together in the bootstrap test (500 replicates) is shown next to the branches. The trees are drawn to scale, with branch lengths in the same units as those of the evolutionary distances used to infer the phylogenetic tree. The analysis involved 25 amino acid sequences. All positions containing gaps and missing data were eliminated. There were a total of 275 positions in the final dataset.

**Figure 4:**
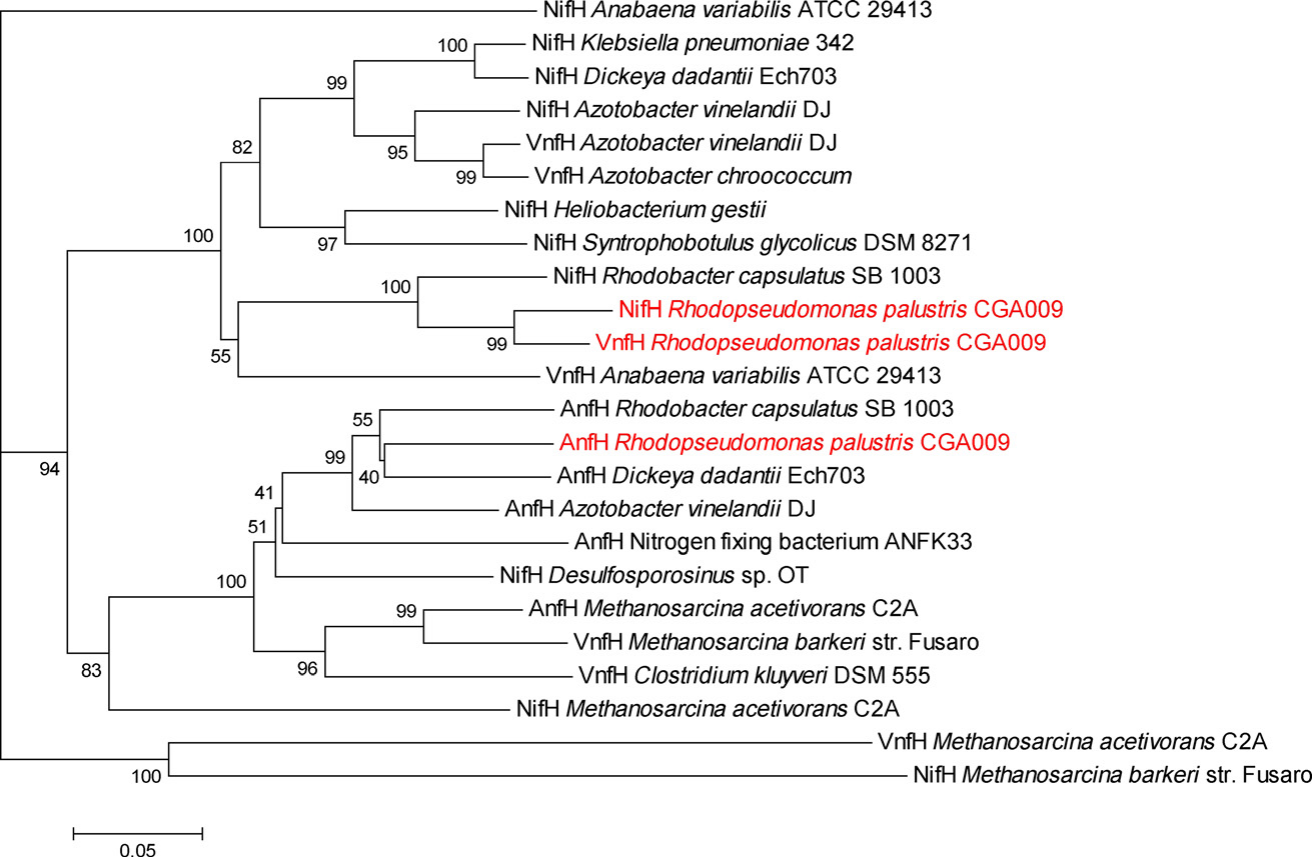
Evolutionary relationships of all three nitrogenase isozymes show disparate origins of the VnfH proteins compared to VnfD (Fig.3). The evolutionary histories of NifH, VnfH, and AnfH were inferred as described for Fig.3. The analysis involved 24 amino acid sequences. All positions containing gaps and missing data were eliminated. There were a total of 224 positions in the final dataset. VnfH in *R. palustris* is more related to the NifH protein of *R. palustris* than to any other NifH homolog.

Fe-nitrogenase enzymes have been shown to be posttranslationally modified in other species of purple nonsulfur bacteria (Lowery et al. 1986; Masepohl et al. 1993). The vanadium isozyme has not been described in other species of purple nonsulfur bacteria and is present in only some strains of *R. palustris*. It is found primarily in *Azotobacter* species, cyanobacteria, and methanogens – phyla that do not have a DraT posttranslational mechanism of nitrogenase control. To probe the evolutionary history of the *R. palustris* alternative nitrogenases, we built phylogenetic trees using neighbor-joining. The tree of the D catalytic subunits of nitrogenases suggests that the three *R. palustris* nitrogenase isozymes (NifD, VnfD, and AnfD) are most closely related to the three corresponding nitrogenase isozymes from *A. vinelandii* DJ (Fig3). A tree of nitrogenase reductase (H) subunits gives a slightly different picture, however, and shows that VnfH from *R. palustris* is most closely related to the *R. palustris* NifH protein, suggesting that VnfH is the product of a gene duplication event (Fig.4).

## Discussion

Our data indicate that the activities of the *R. palustris* alternative nitrogenase enzymes are inactivated when cells are exposed to ammonium, and this is likely mediated by a posttranslational modification of the cognate dintirogenase reductases by ADP-ribosylation. Of 16 closely related and recently sequenced strains of *R. palustris*, all have Mo- and Fe-nitrogenase, but three lack V-nitrogenases (https://img.jgi.doe.gov/cgi-bin/er/main.cgi). Slightly more distantly related strains of *R. palustris* all lack V-nitrogenase (Oda et al. 2008). This suggests that *R. palustris* may have acquired its V nitrogenase genes by horizontal gene transfer. Regardless of the evolutionary origin of the structural genes for the alternative nitrogenases, all three nitrogenases in *R. palustris* CGA009 rely on several common components for production of metal cofactors and their insertion into the holoenzyme (Schuddekopf et al. 1993; Oda et al. 2005). The V-nitrogenase is a more efficient enzyme for nitrogen fixed per reducing power used than the heterometal free Fe-nitrogenase and is also more active at lower temperatures than the Mo-nitrogenase (Miller and Eady 1988; Eady 1996). In *R. palustris*, the structural genes for both alternative nitrogenases are tightly regulated such that they are only expressed when the Mo-nitrogenase is ineffective at meeting the fixed nitrogen needs of cells (Oda et al. 2005).

While VnfD, AnfD, and NifD from a wide variety of organisms fall into clades separated by the metal they use at the active site (Fig.3), the dinitrogenase reductase proteins (VnfH, AnfH, and NifH) do not separate similarly in our analysis (Fig.4). These results confirm previous analyses, (Raymond et al. 2004; Young 2005). The *R. palustris* VnfH and NifH proteins appear more related to each other than to any other dinitrogenase reductase protein analyzed. This suggests that VnfH in *R. palustris* is the product of a gene duplication event. A possible scenario for this gene duplication is as follows. *R. palustris* CGA009 expanded its nitrogen fixation abilities by horizontally acquiring the *vnf* gene cluster, which includes structural as well as some accessory genes, perhaps from a species similar to *A. vinelandii* (Bishop et al. 1980; Hales et al. 1986). The acquired VnfH would be approximately 71% identical and 86% similar to *R. palustris* NifH and possibly be less efficiently modified by DraT2, even though it has an identical site of modification (Fig.2). To bring V-nitrogenase under tight regulatory control, recombination between the *vnf* and *nif* gene clusters could result in duplication of *nifH*. The ability of VnfH to substitute for NifH has been demonstrated in *A. vinelandii* (Chatterjee et al. 1997).

Here, we have presented evidence that all three nitrogenase isozymes from *R. palustris* are subject to posttranslational modification by ADP-ribosylation. This suggests for maximum hydrogen production, the posttranslational regulation mechanism must be removed in strains of *R. palustris* engineered to express any of the three nitrogenases.
